# Correction: A disordered encounter complex is central to the yeast Abp1p SH3 domain binding pathway

**DOI:** 10.1371/journal.pcbi.1013201

**Published:** 2025-06-18

**Authors:** Gabriella J. Gerlach, Rachel Carrock, Robyn Stix, Elliott J. Stollar, K. Aurelia Ball

S1 Fig is incorrect. Please see the correct S1 Fig below.

## Supporting information

S1 Fig Pairwise distances used in the determination of whether the encounter simulation has reached the bound state. The distances K(3)-Y10, P(2)-V55, P(0)-Y54, and K(-3)-K38 were used in the seg1 simulations and all seven distances were used in the ArkA simulations. The pairwise distance in the NMR ranges from 8.73 to 8.99 Å.
(PNG)

## References

[1] Gerlach GJ, Carrock R, Stix R, Stollar EJ, Ball KA. A disordered encounter complex is central to the yeast Abp1p SH3 domain binding pathway. PLoS Comput Biol. 2020;16(9):e1007815. doi: 10.1371/journal.pcbi.1007815 32925900 PMC7514057

