# Supplementary material for: A disordered encounter complex is central to the yeast Abp1p SH3 domain binding pathway
Source: PLoS Comput Biol. 2020 Sep 14;16(9):e1007815. doi: 10.1371/journal.pcbi.1007815 (PMC7514057; doi:10.1371/journal.pcbi.1007815)
Supplement: S1 Text — (PDF) [file pcbi.1007815.s001.pdf]

S1 Text. Seg1 binding results.

Seg1 contains the class II PxxPx+ motif, which is almost the reverse of the class I +xxPxxP motif. With this pseudo-palandromic motif, seg1 is likely to be able to bind in two different orientations, and experimental data shows that seg1 binds the domain with a lower affinity than ArkA [38]. We wanted to test whether the binding pathway differs for the shorter peptide in order to examine the role of seg1 and seg2 in binding. To initiate the seg1 binding simulations, we divided the unbound ArkA ensemble into two states based on the seg1 dihedral RMSD with a cutoff of 38.1˚ (S9 Fig) and ran 25 independent seg1 binding simulations for 500 ns each, starting from each of the two states (Table 1). We found that seg1 also occupies an encounter complex before reaching the fully engaged state, but it spends more time in both forward and reverse orientations within the encounter complex ensemble than the ArkA peptide does. It may be that the short seg1 peptide is not a good proxy for how seg1 behaves as part of the longer peptide.

In the seg1 simulations, the encounter complex was ~10 times more likely to transition to the unbound state than the fully engaged state (we observed 49 transitions to unbound and only 4 to fully engaged from the encounter complex). ArkA is only ~5 times more likely to transition to the unbound state than the fully engaged state when in the encounter complex. This indicates that the barrier between unbound and the encounter complex is lower for seg1 than for ArkA, which is supported by the higher k_1 rate constant for seg1 in our simulations. Since the seg1 peptide has fewer groups that can interact nonspecifically with the domain in the encounter complex ensemble, it is easier for it to transition to a state where it will dissociate completely.
