# Supplementary material for: A disordered encounter complex is central to the yeast Abp1p SH3 domain binding pathway
Source: PLoS Comput Biol. 2020 Sep 14;16(9):e1007815. doi: 10.1371/journal.pcbi.1007815 (PMC7514057; doi:10.1371/journal.pcbi.1007815)
Supplement: S1 Table — (PDF) [file pcbi.1007815.s002.pdf]

S1 Table. Temperatures (in Kelvin) used in the unbound ArkA replica exchange simulations.

The temperature used for analysis is in bold.

290.00	330.30	376.20
292.37	332.99	379.27
294.76	335.71	382.36
297.16	338.45	385.49
**299.59**	341.22	388.63
302.03	344.00	391.81
304.50	346.81	395.01
306.99	349.65	398.23
309.49	352.50	401.48
312.02	355.38	404.76
314.57	358.28	408.07
317.14	361.21	411.40
319.73	364.15	414.76
322.34	367.13	418.14
324.97	370.13	421.56
327.62	373.15	425.00
