# Supplementary material for: A disordered encounter complex is central to the yeast Abp1p SH3 domain binding pathway
Source: PLoS Comput Biol. 2020 Sep 14;16(9):e1007815. doi: 10.1371/journal.pcbi.1007815 (PMC7514057; doi:10.1371/journal.pcbi.1007815)
Supplement: S2 Table — (PDF) [file pcbi.1007815.s003.pdf]

S2 Table. Summary of volume, concentration, and binding frequency for the two binding simulations.

	Volume (Å^3)	Concentration (mM)	Binding frequency (s^{-1})
ArkA	437,360	3.80	2.4×10^5
seg1	403,200	4.12	1.9×10^5
