# Supplementary material for: A disordered encounter complex is central to the yeast Abp1p SH3 domain binding pathway
Source: PLoS Comput Biol. 2020 Sep 14;16(9):e1007815. doi: 10.1371/journal.pcbi.1007815 (PMC7514057; doi:10.1371/journal.pcbi.1007815)
Supplement: S3 Table — (PDF) [file pcbi.1007815.s004.pdf]

S3 Table. Summary of water box dimensions for each simulated system.

	Dimensions (Å)		
Bound simulations	49	49	49
Unbound (extended)	63	63	63
Unbound (NMR)	59	59	59
ArkA binding	71	77	80
Seg1 binding	60	80	84
