# Supplementary figures and images for: A disordered encounter complex is central to the yeast Abp1p SH3 domain binding pathway
Source: PLoS Comput Biol. 2020 Sep 14;16(9):e1007815. doi: 10.1371/journal.pcbi.1007815 (PMC7514057; doi:10.1371/journal.pcbi.1007815)

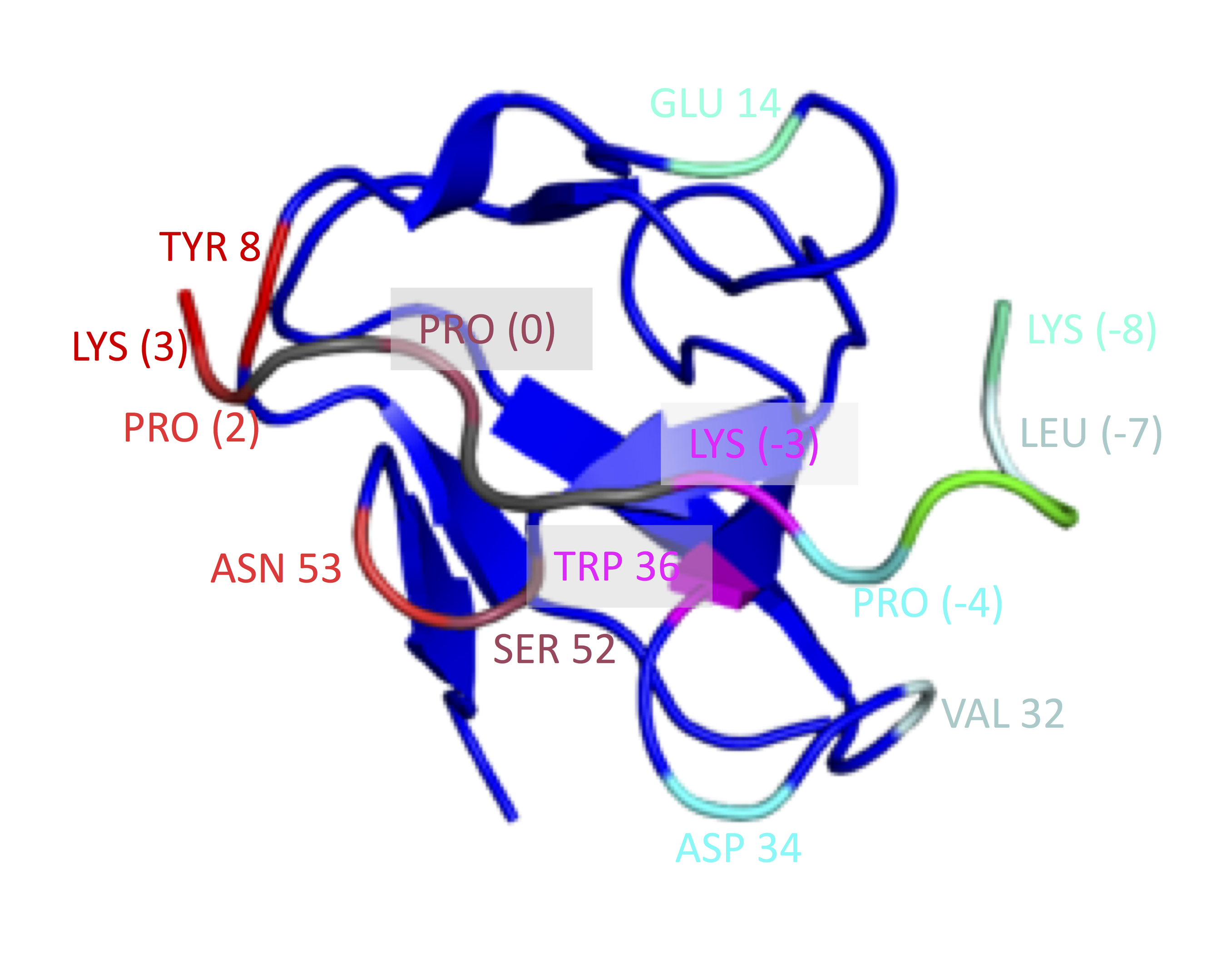

Supplement: S1 Fig — The pairs in red and magenta where used for both the seg1 and ArkA1 simulations and the cyan pairs where added for the ArkA simulations. The distances were determined based on the SH3 residues whose chemical shifts where used to determine binding in NMR experiments. The pairwise binding surface distance in the NMR structures ranges from 7.25 to 7.62 Å. (TIF) [file pcbi.1007815.s005.tif]

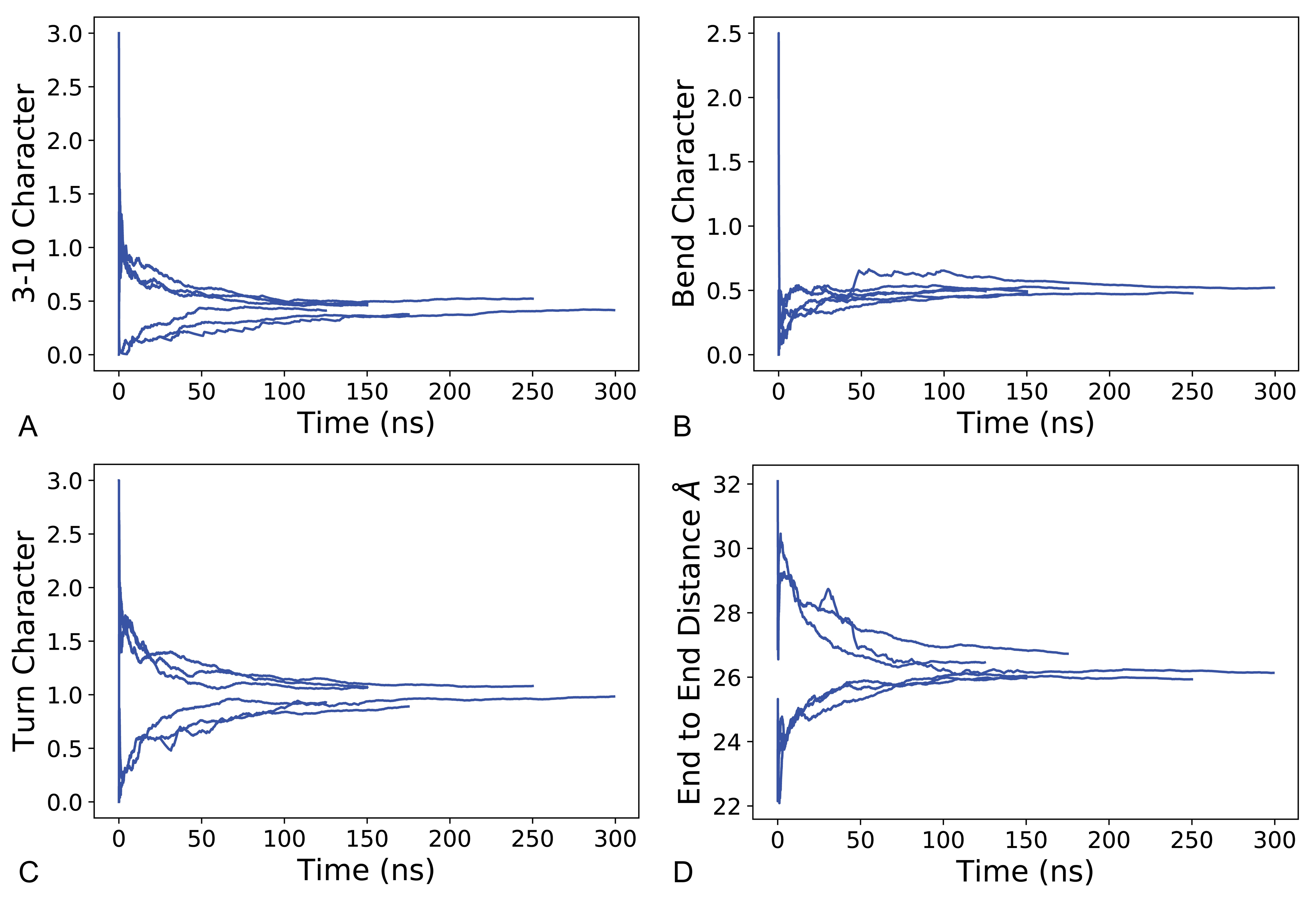

Supplement: S2 Fig — ArkA structural measures plotted vs. simulation time for each of the independent REMD simulations. The first 50 ns of each independent simulation was removed before analysis. (TIF) [file pcbi.1007815.s006.tif]

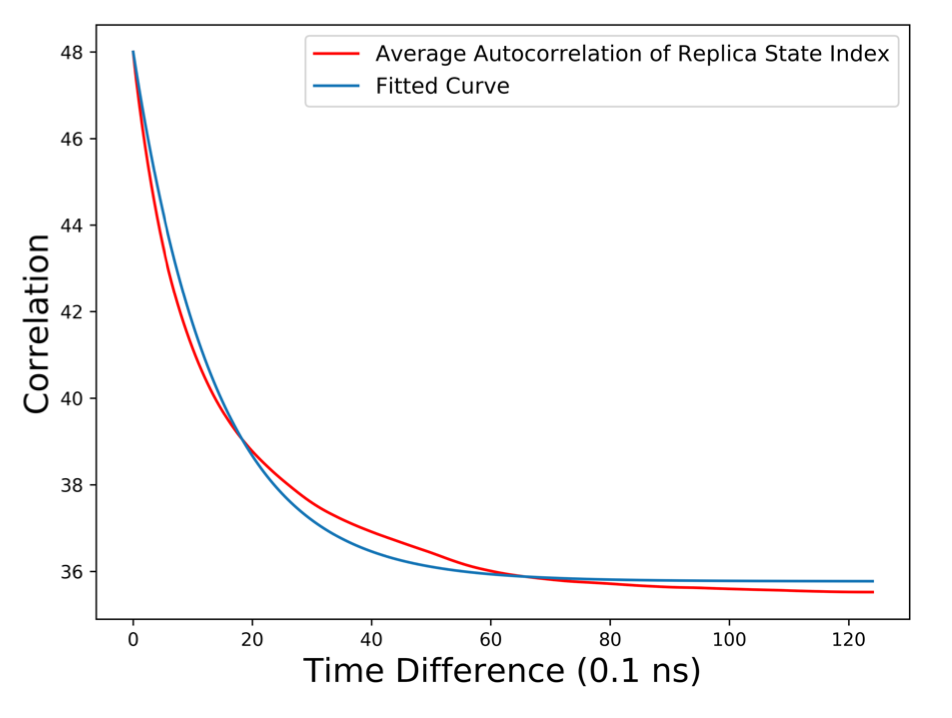

Supplement: S3 Fig — (PNG) [file pcbi.1007815.s007.png]

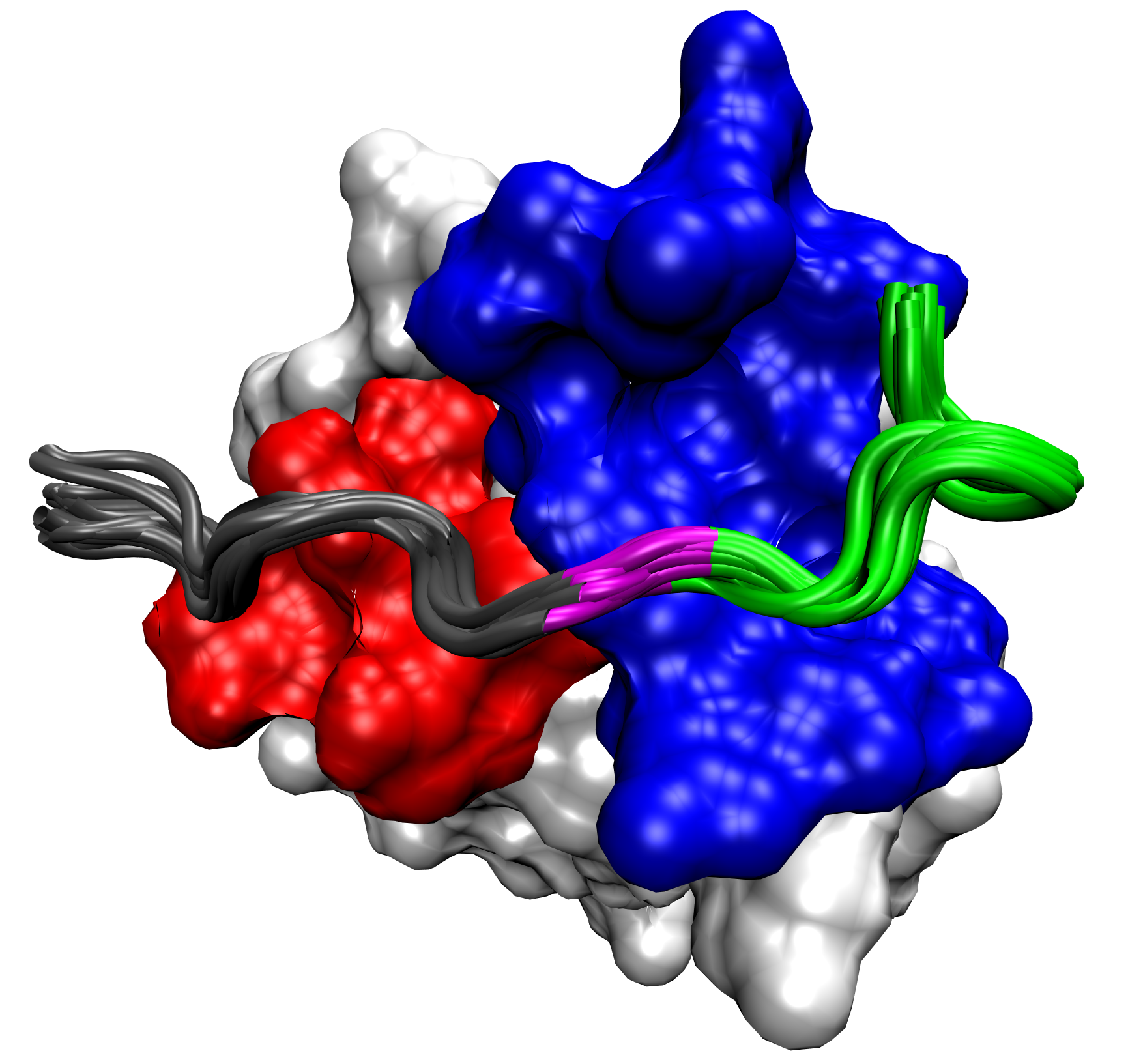

Supplement: S4 Fig — (TIFF) [file pcbi.1007815.s008.tiff]

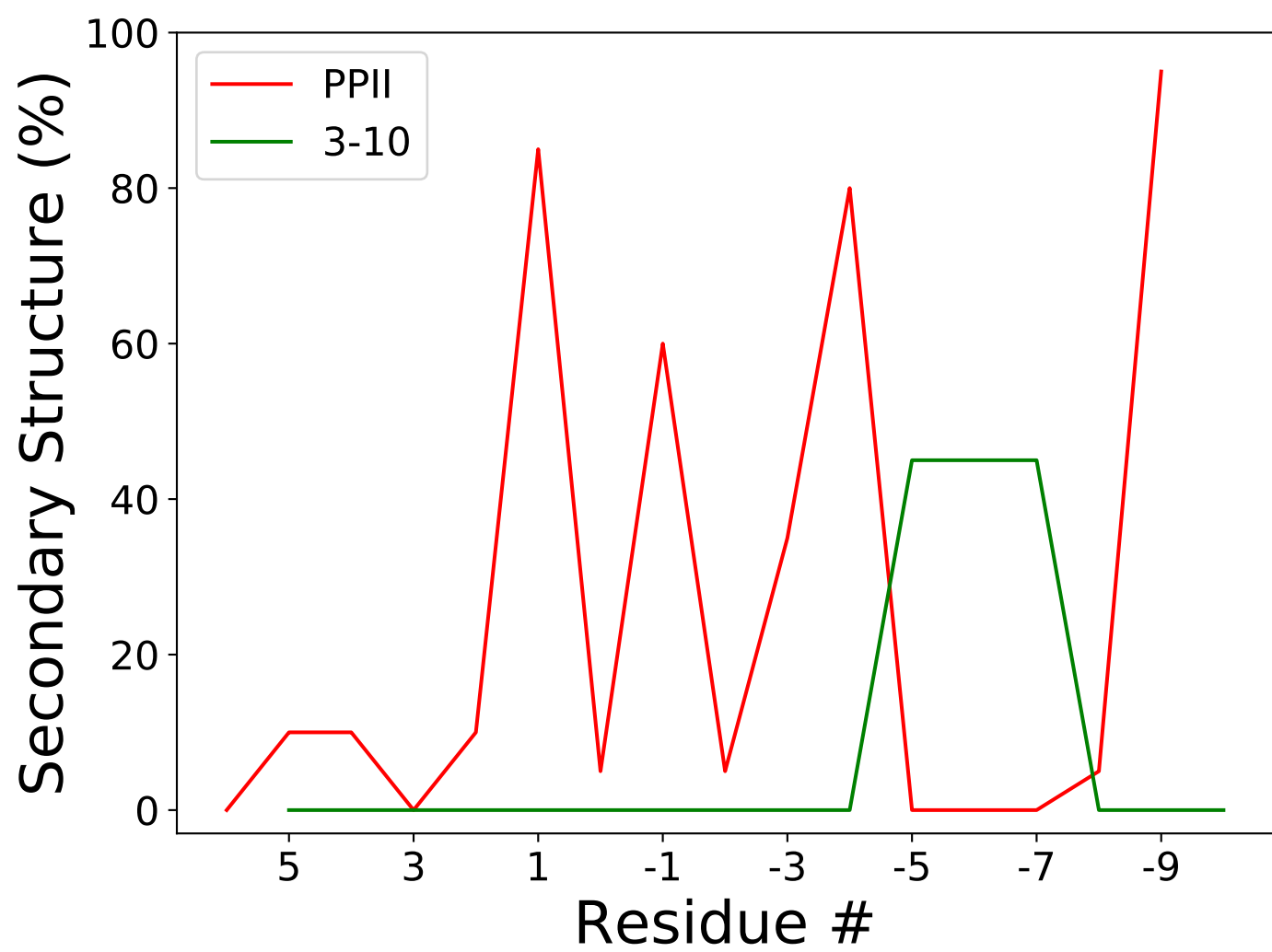

Supplement: S5 Fig — (PDF) [file pcbi.1007815.s009.pdf]

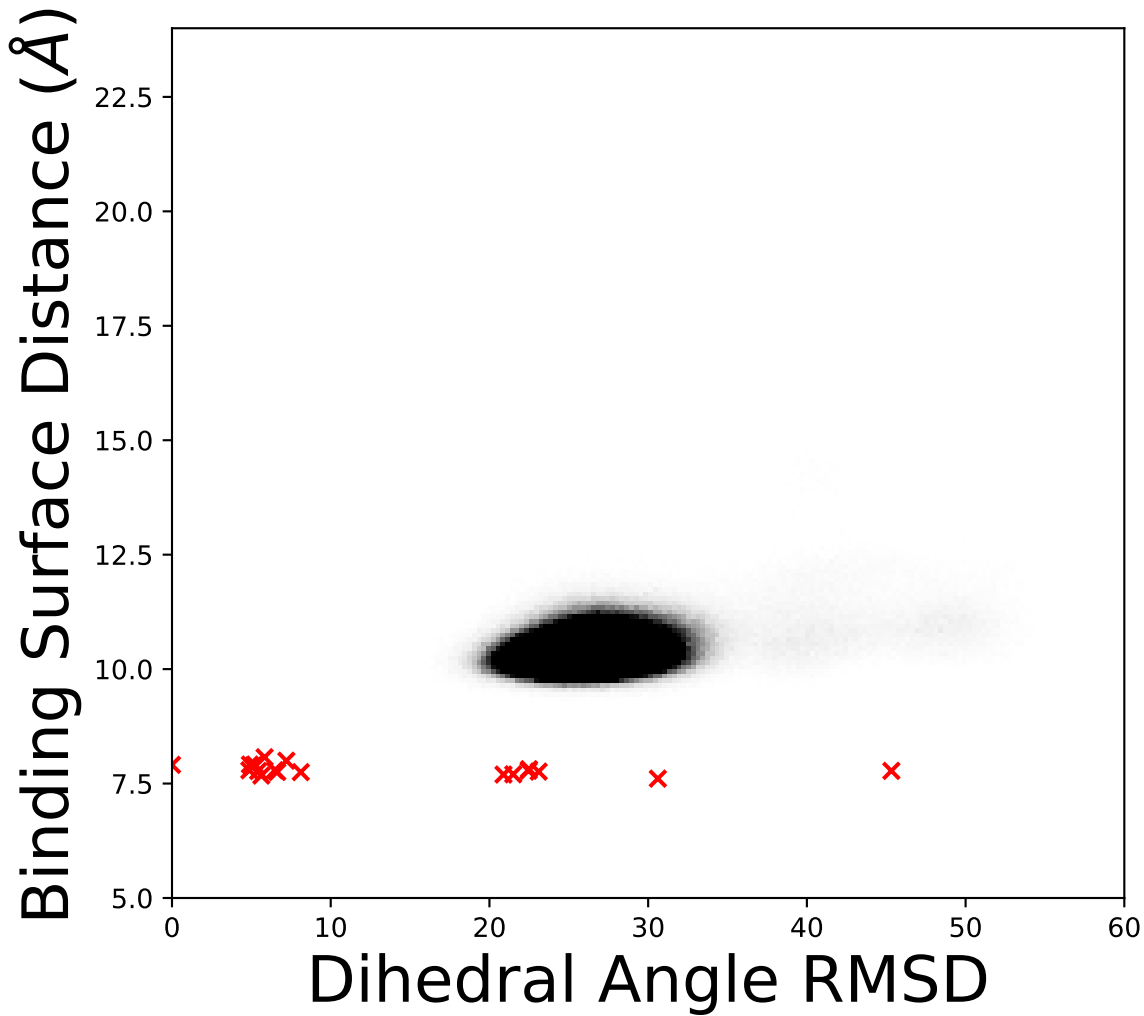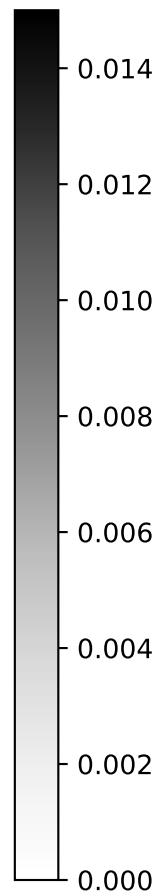

Supplement: S6 Fig — The distance between ArkA and the binding surface of AbpSH3 is graphed against the dihedral angle RMSD for the bound simulations with the 20 NMR structures shown as red x’s. The NMR structures all have low binding surface distances, but cover a range of different dihedral angle RMSD values. Darker shading indicates a larger fraction of the total bound simulation ensemble, as indicated by the color bar. (PDF) [file pcbi.1007815.s010.pdf]

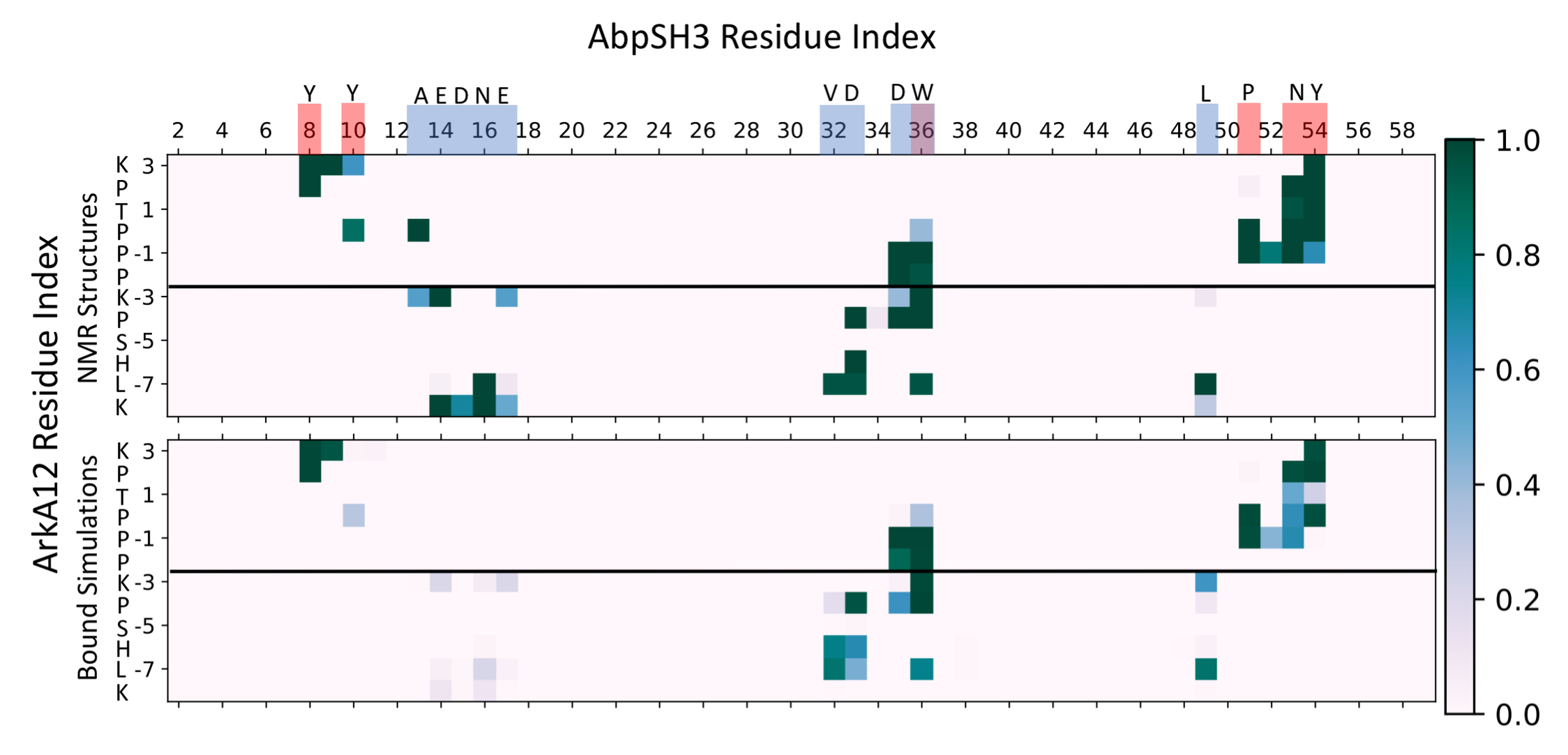

Supplement: S7 Fig — The darker squares indicate more of the ensemble with that contact. The red and blue squares along the AbpSH3 residue index show which amino acids are in SI and SII, respectively. The black line indicates the separation of seg1 and seg2, and the single letter amino acid codes are included for ArkA and the residues in SI and SII. (TIF) [file pcbi.1007815.s011.tif]

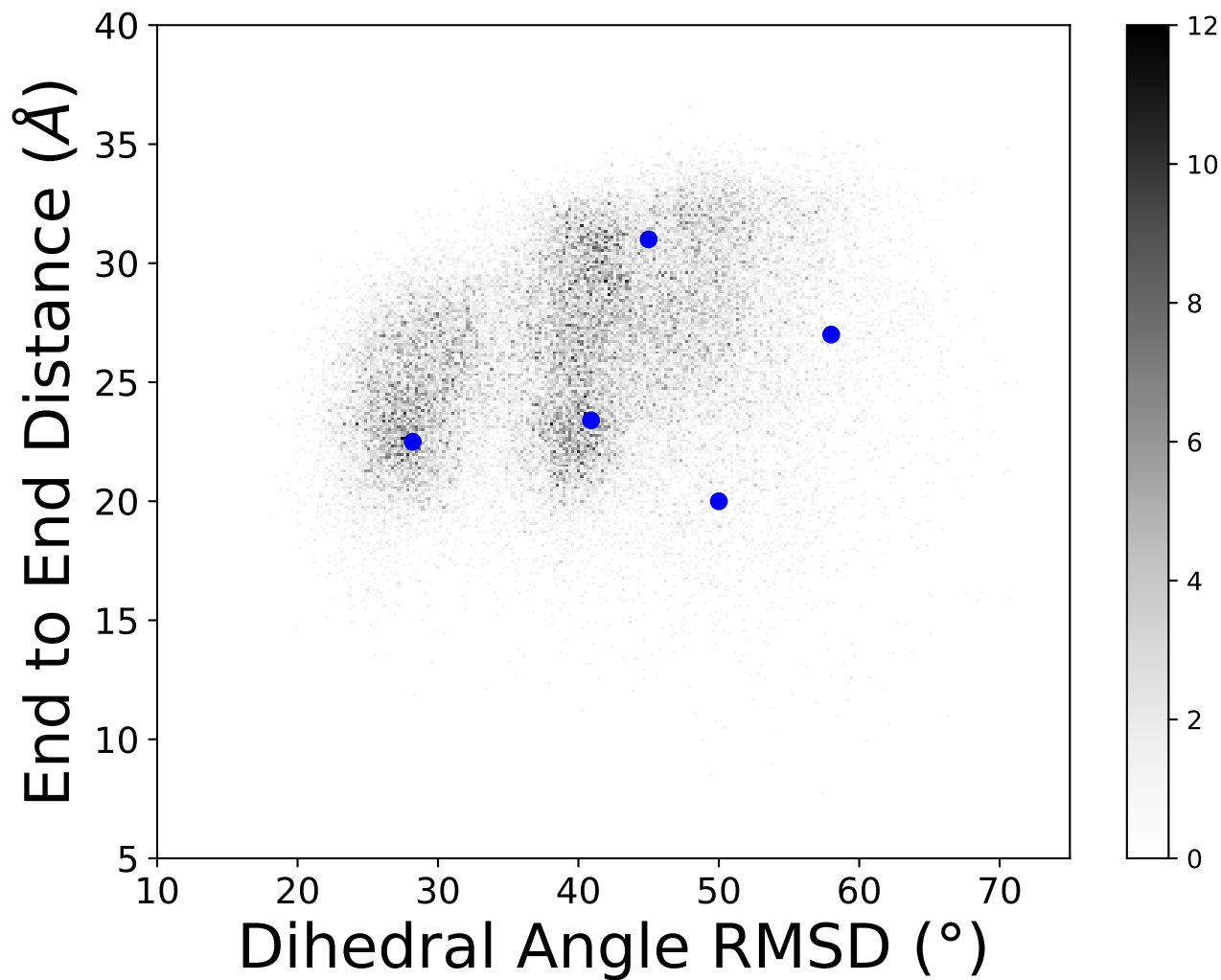

Supplement: S8 Fig — The conformational ensemble of unbound ArkA from the REMD simulations is plotted in terms of end-to-end distance and dihedral angle RMSD, with starting structures for ArkA binding simulations indicated by blue circles. End-to-end distance is the distance between the C and N-terminal ends of ArkA and dihedral angle RMSD is calculated only for ArkA with the lowest energy NMR structure (2RPN) as the reference [72]. (PDF) [file pcbi.1007815.s012.pdf]

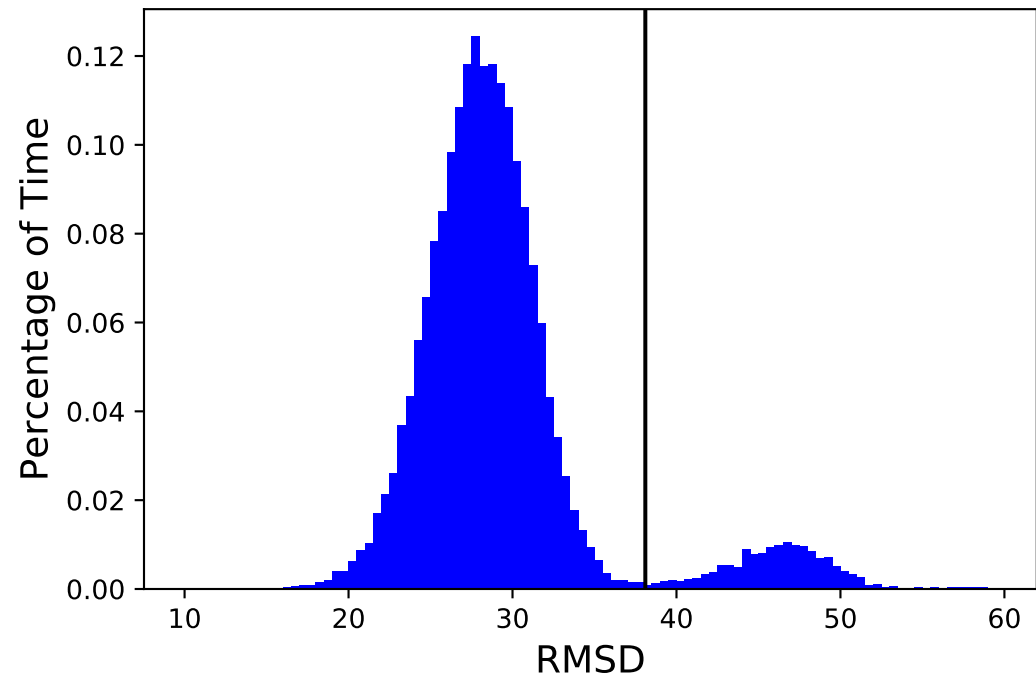

Supplement: S9 Fig — The vertical line at 38.1° indicates the cutoff that was determined between the two states (native and nonnative conformations). (PDF) [file pcbi.1007815.s013.pdf]

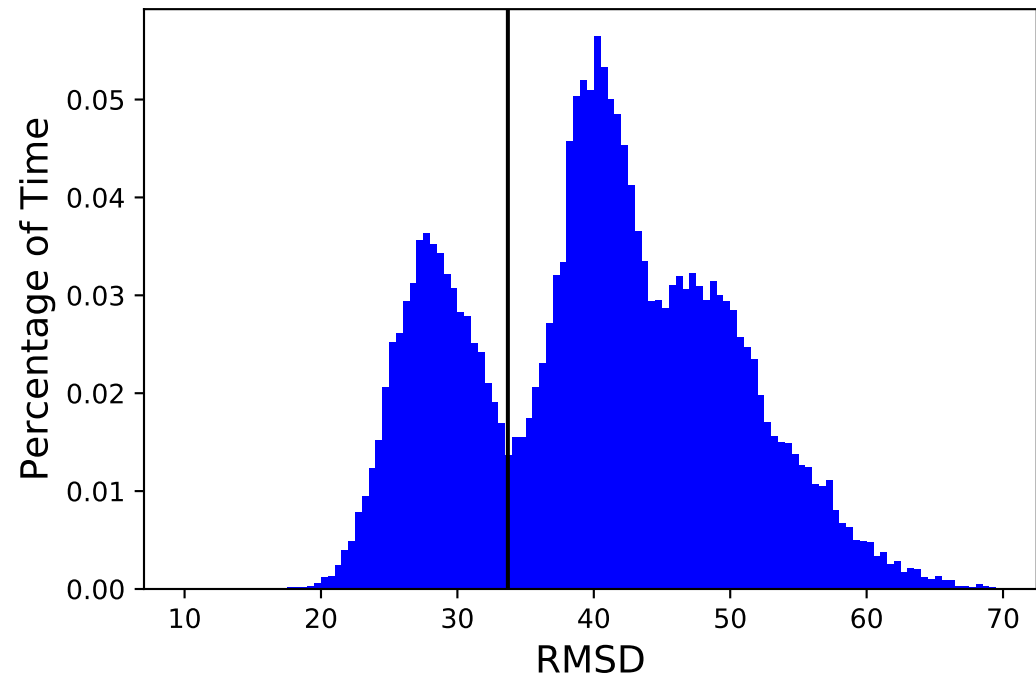

Supplement: S10 Fig — The vertical line at 33.7° indicates the cutoff that was determined between the native folded and nonnative states. (PDF) [file pcbi.1007815.s014.pdf]

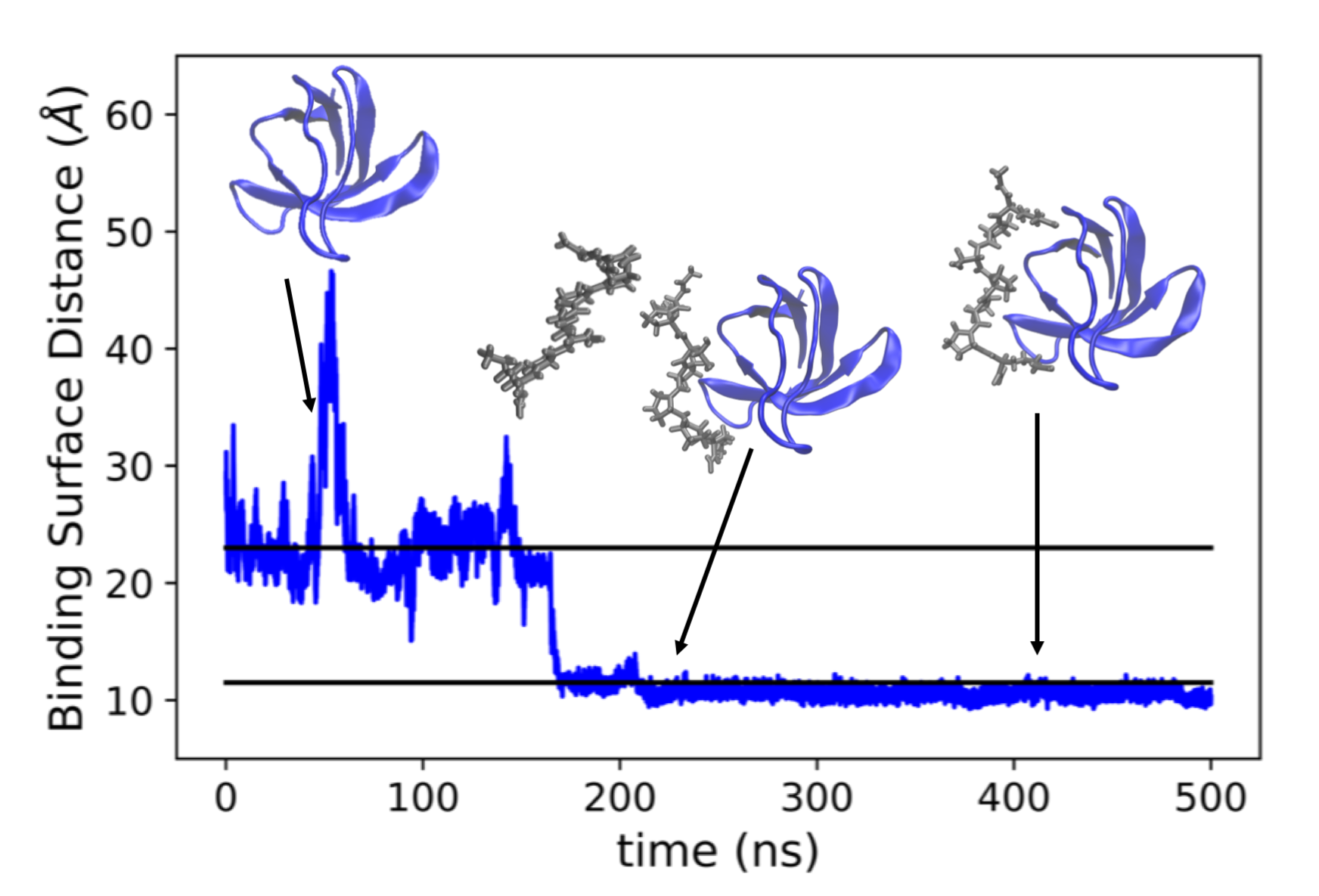

Supplement: S11 Fig — The black lines correspond to our definition of the encounter complex (23 Å) and the fully engage complex (11.5 Å). (TIF) [file pcbi.1007815.s015.tif]

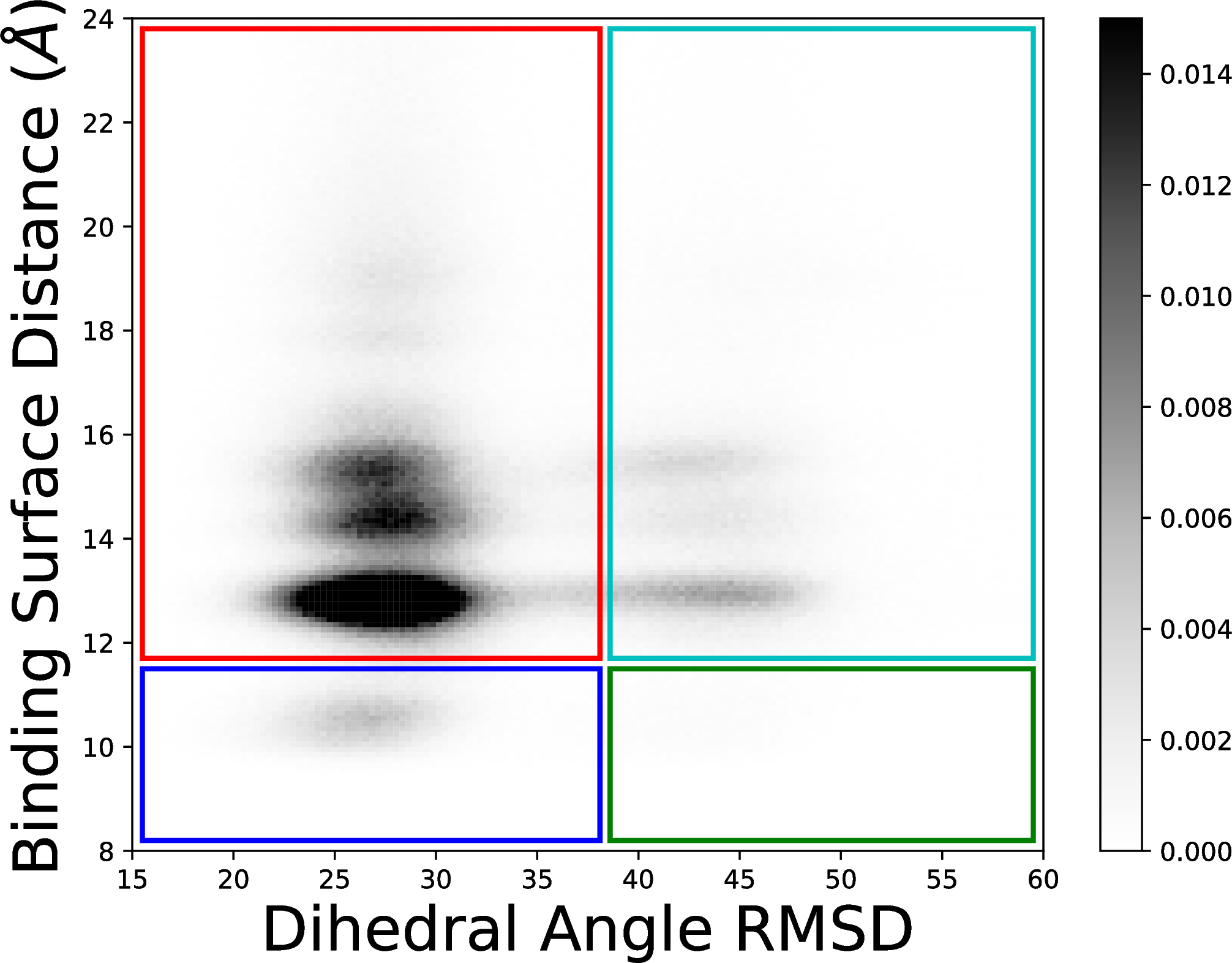

Supplement: S12 Fig — Darker shading indicates a larger fraction of the total ensemble, as indicated by the color bar. Colored boxes partition the ensemble into four states: folded and fully engaged (blue), unfolded and fully engaged (green), folded and encounter (red), unfolded and encounter (cyan). (TIF) [file pcbi.1007815.s016.tif]

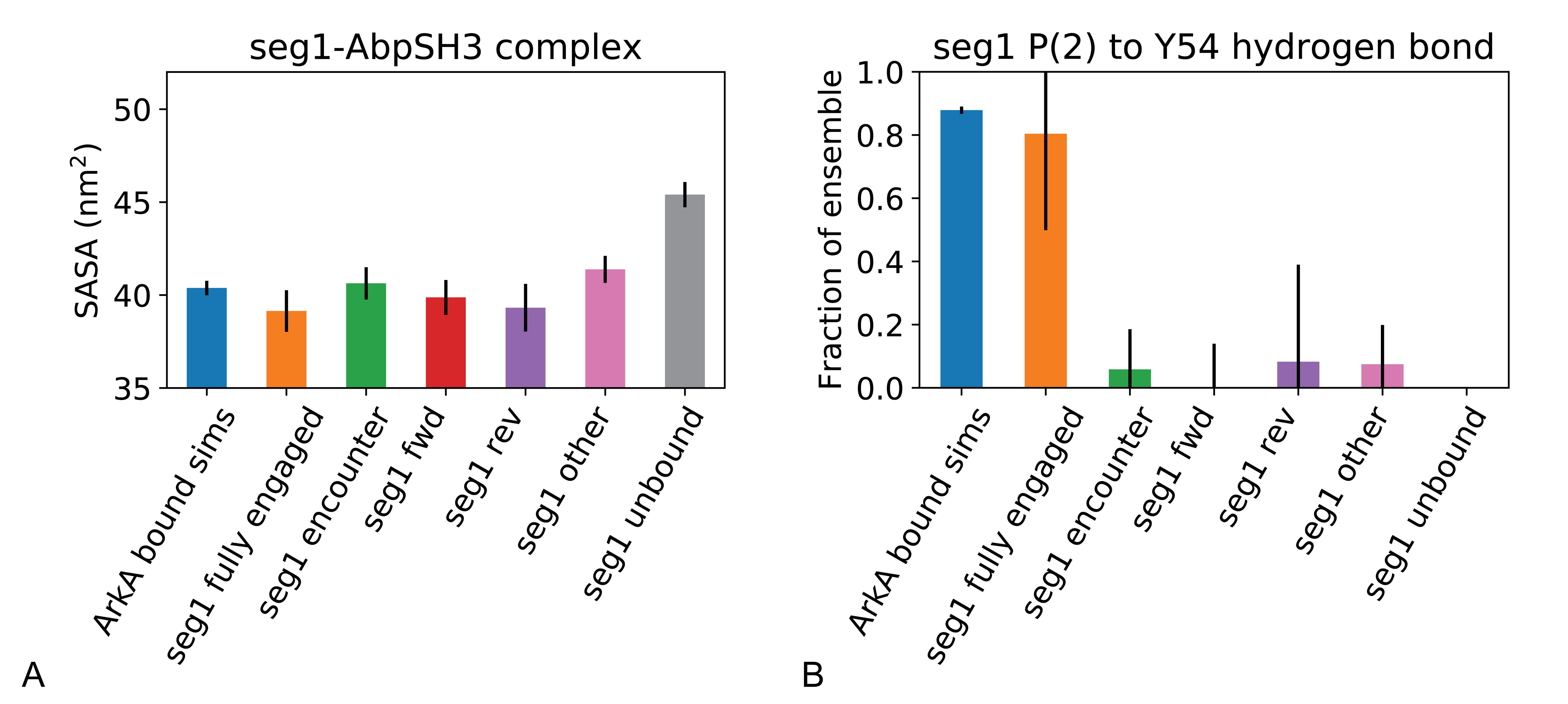

Supplement: S13 Fig — Solvent accessible surface area (A) and occupancy of the P(2) to Y54 hydrogen bond (B) for the seg1-AbpSH3 complex in different states. The first bar on the plot represents the solvent accessible surface area in bound simulations. Error bars represent the standard deviation between independent simulations. (TIF) [file pcbi.1007815.s017.tif]

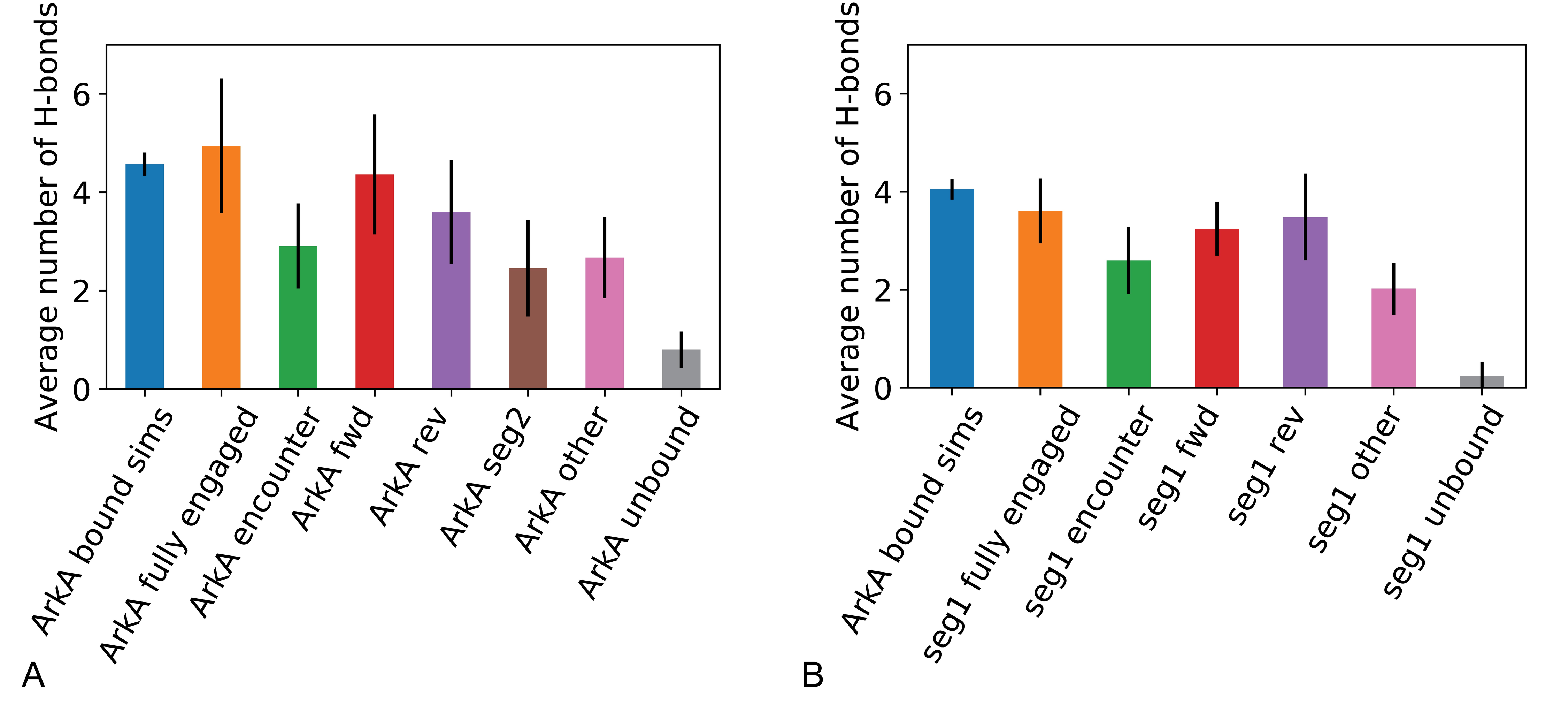

Supplement: S14 Fig — Average number of hydrogen bonds (or salt bridges) between AbpSH3 and the ArkA peptide for ArkA (A) and seg1 (B) in the bound simulations (first bar) and binding simulations by state of the complex. Error bars represent the standard deviation between independent simulations. (TIF) [file pcbi.1007815.s018.tif]

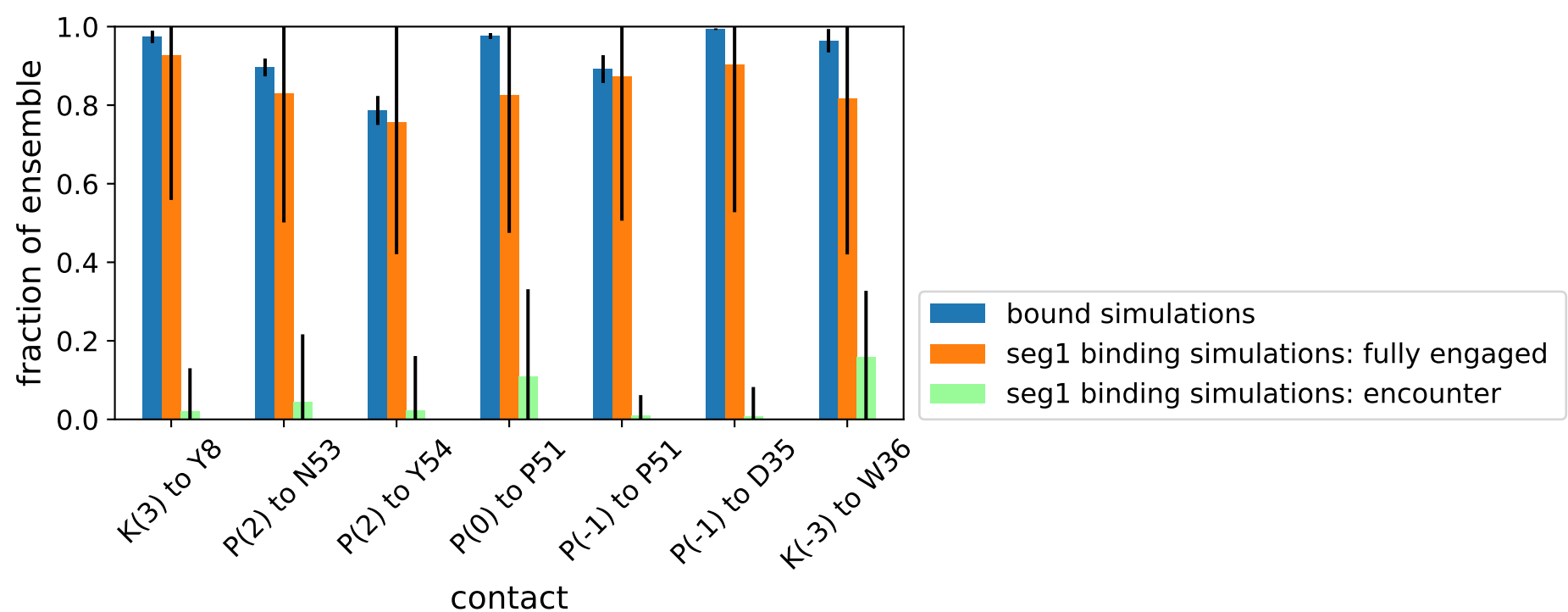

Supplement: S15 Fig — Hydrophobic contacts were selected from those hydrocarbon groups that are closest together in the NMR structural ensemble (2RPN) [72]. Contacts were defined based on a 6 Å cutoff distance between hydrocarbon groups. Error bars represent the standard deviation between independent simulations. (PDF) [file pcbi.1007815.s019.pdf]

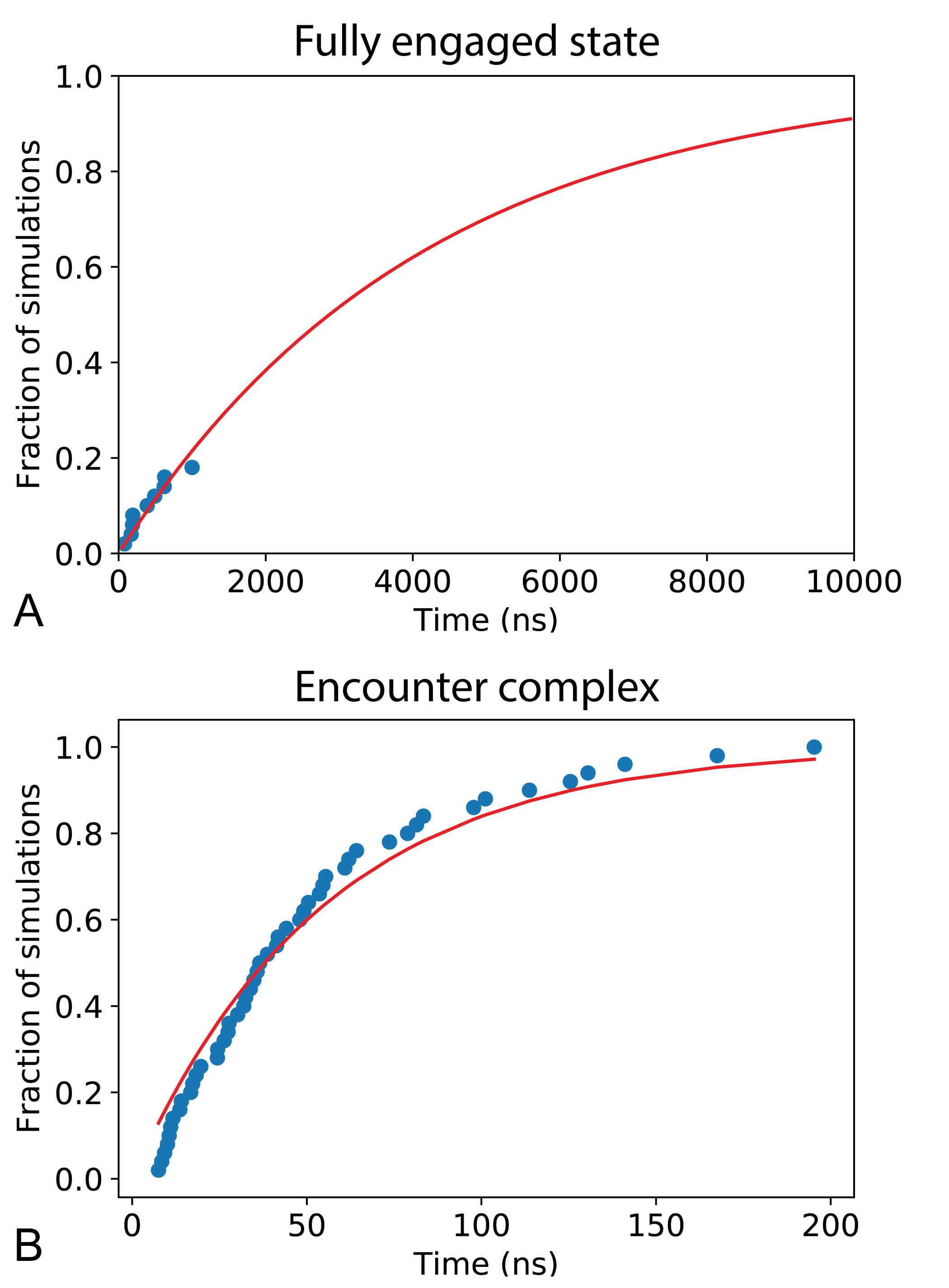

Supplement: S16 Fig — Fit of TCDF curve (red line) to time to formation data (blue circles) for the fully engaged ArkA complex (A) and for the ArkA encounter complex (B) from ArkA binding simulations. These fits were used to determine kon and k1 respectively. (TIF) [file pcbi.1007815.s020.tif]

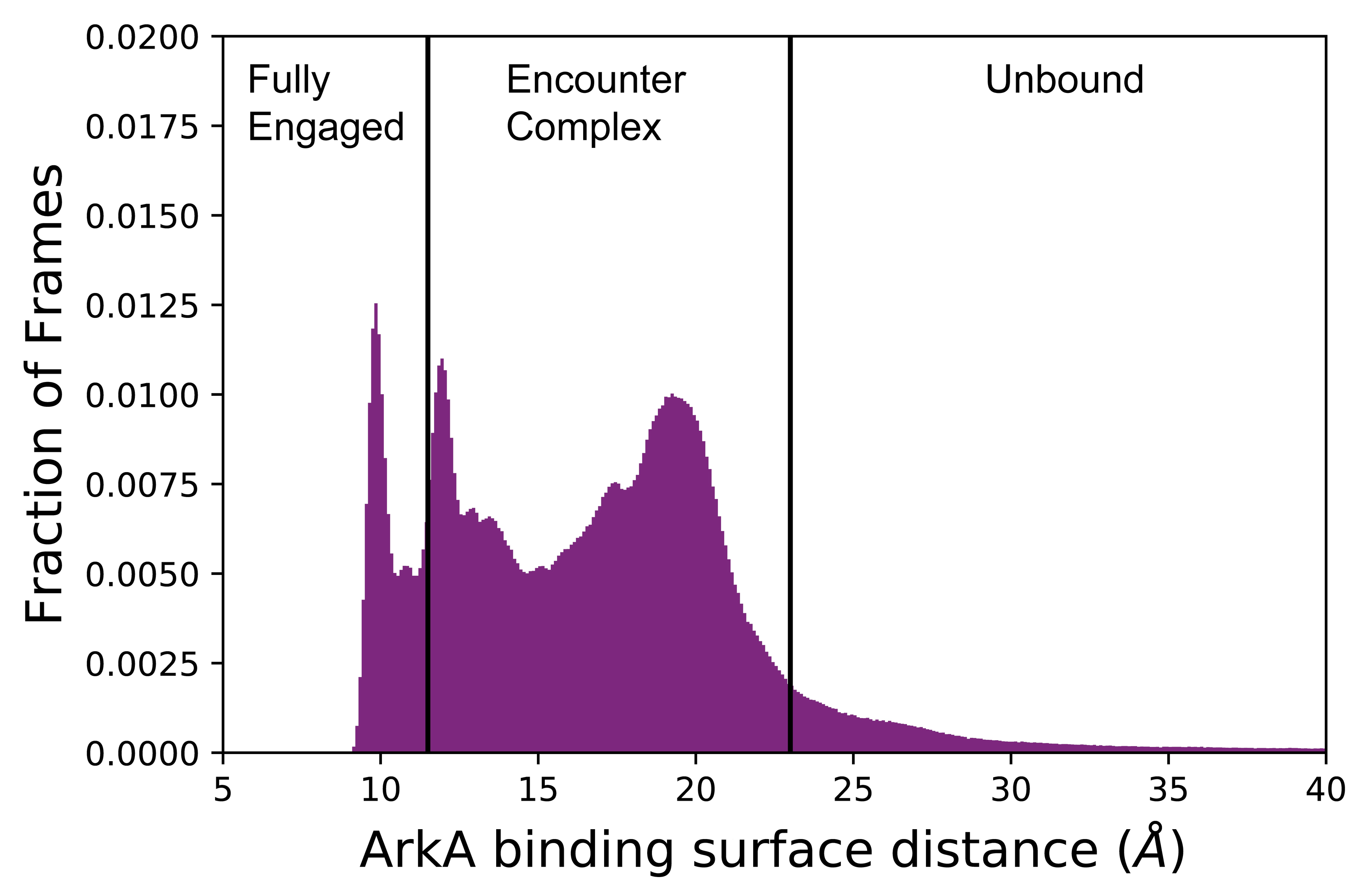

Supplement: S17 Fig — States left of the vertical line at 11.5 Å are classified as fully engaged. States between the vertical line at 11.5 Å and the one at 23 Å are classified as the encounter complex. States right of the vertical line at 23 Å are classified as unbound. There is a clear population decrease between the fully engaged and encounter complexes, indicating a free energy barrier. It is important to note that these binding simulations do not fully sample the fully engaged state or the barrier between fully engaged and encounter, so this histogram cannot be considered an equilibrium ensemble. There is no clear barrier between the unbound state and encounter complex, indicating that formation of the encounter complex from the unbound state is downhill in free energy. Within the encounter complex, the binding surface distance reaction coordinate reveals two populations. The population between 11.5 and 15 Å is 32% of the entire encounter complex ensemble and contains almost all of the forward encounter states (67.1% other, 28.9% forward, 4.0% segment 2 only). The population between 15 and 23 Å is 68% of the encounter complex ensemble and contains all of the reverse encounter states (82.7% other, 12.8% reverse, 2.8% segment 2 only, 0.6% forward). (TIF) [file pcbi.1007815.s021.tif]

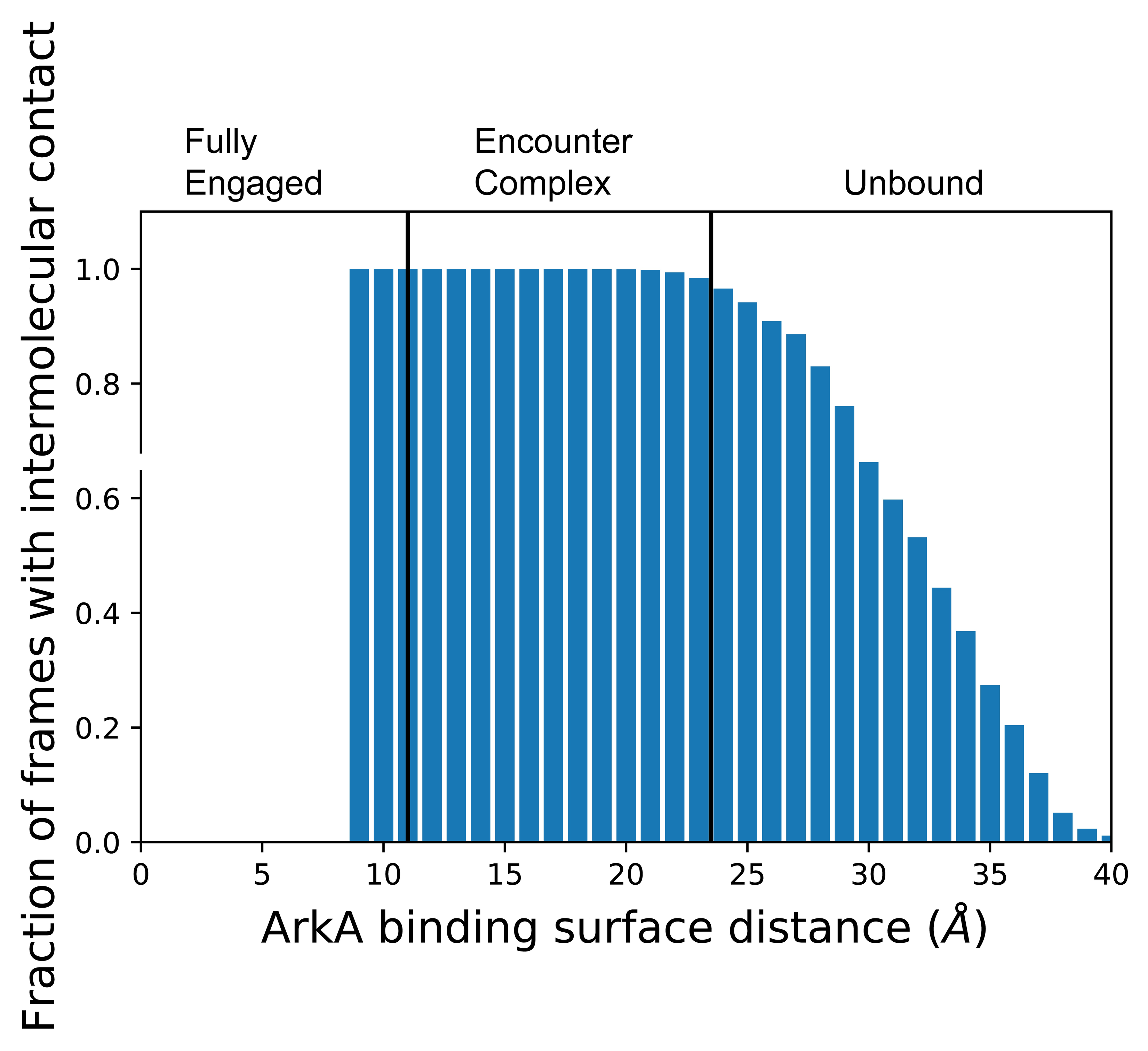

Supplement: S18 Fig — The blue bars represent the fraction of frames that have at least one intermolecular contacts between ArkA and AbpSH3 at each bin along the binding surface distance reaction coordinate. The vertical black line at 11.5 Å represents the division between the fully engaged and encounter complex states, while the vertical black line at 23 Å represent the division between the encounter complex and the unbound state. While 100% of the fully engaged structures and 99.9% of the encounter complex structures have at least one intermolecular contact between ArkA and AbpSH3, 34% of the unbound structures have no contacts between ArkA and AbpSH3. (TIF) [file pcbi.1007815.s022.tif]

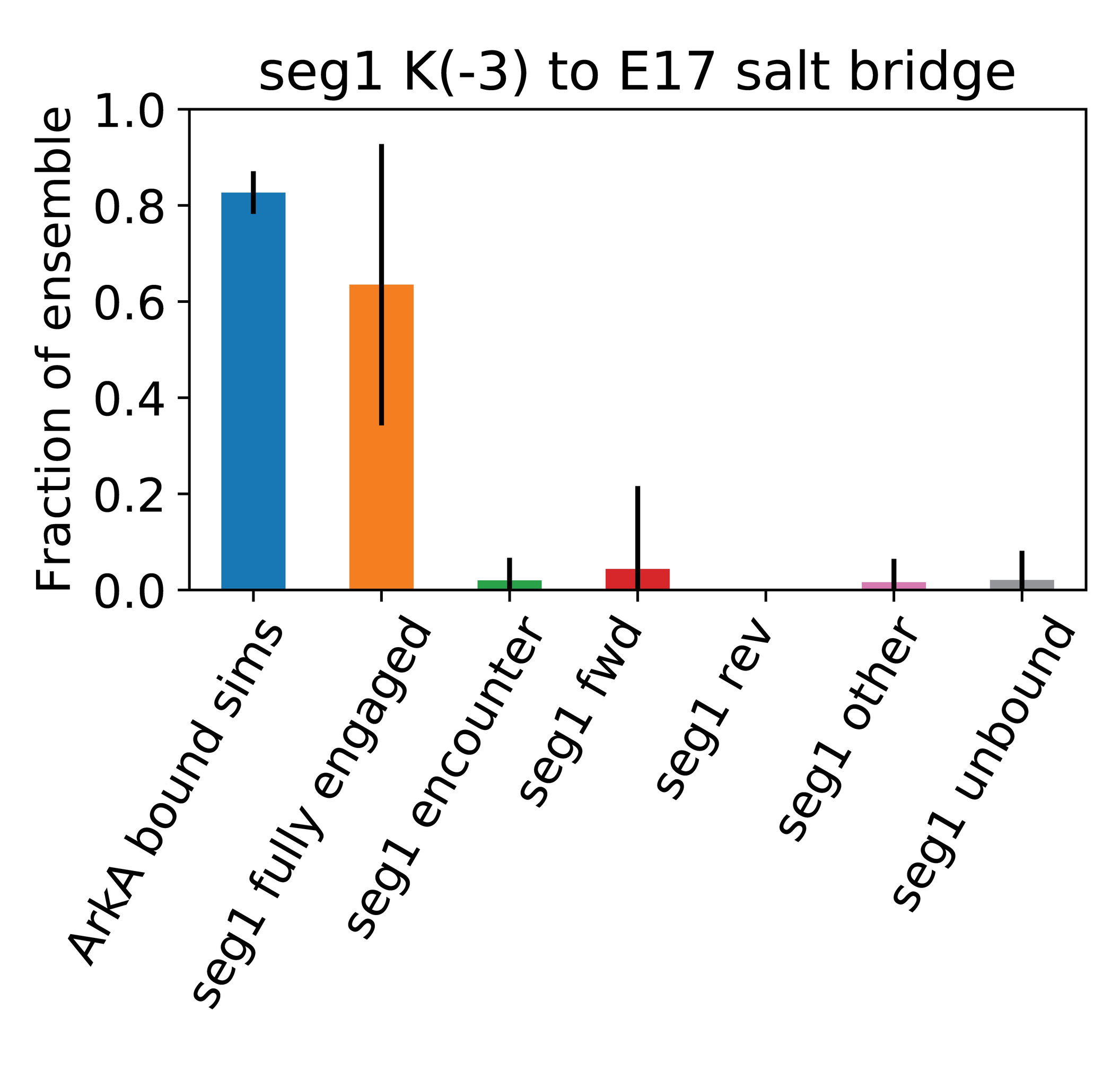

Supplement: S19 Fig — The first bar on the plot represents the salt bridge occupancy in bound simulations. Error bars represent the standard deviation between independent simulations. (TIF) [file pcbi.1007815.s023.tif]

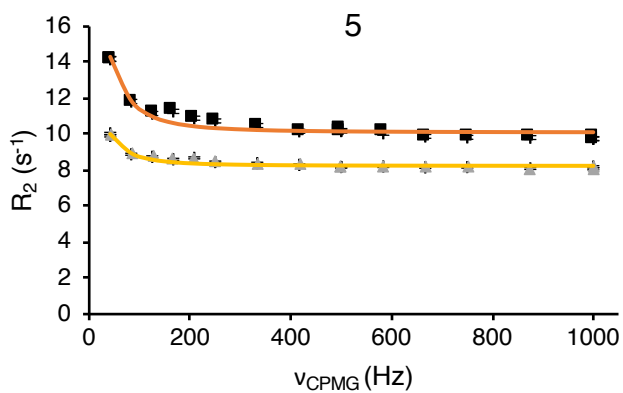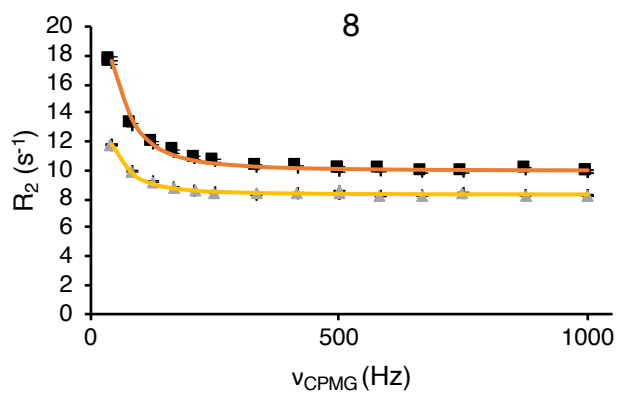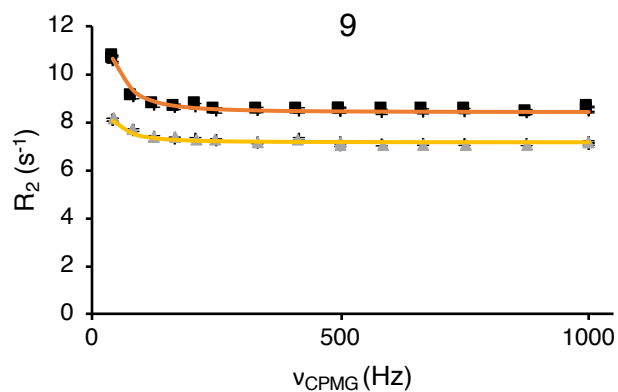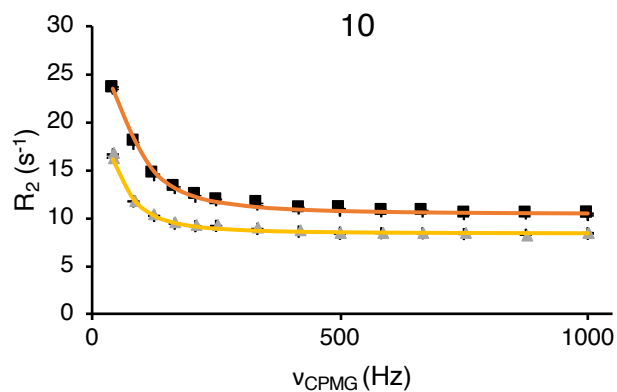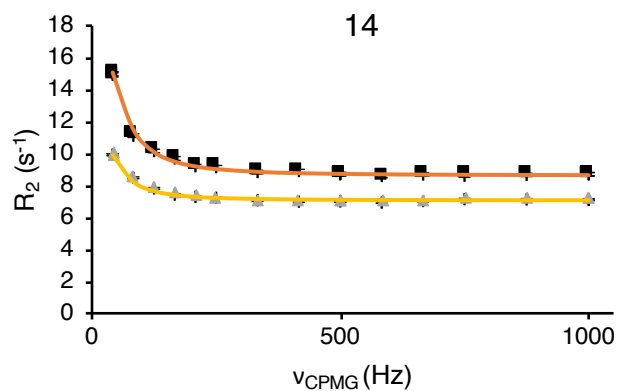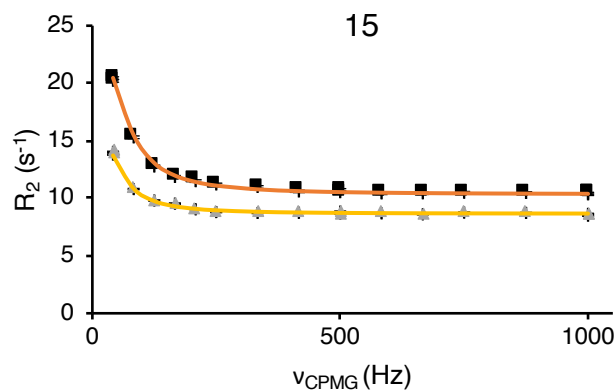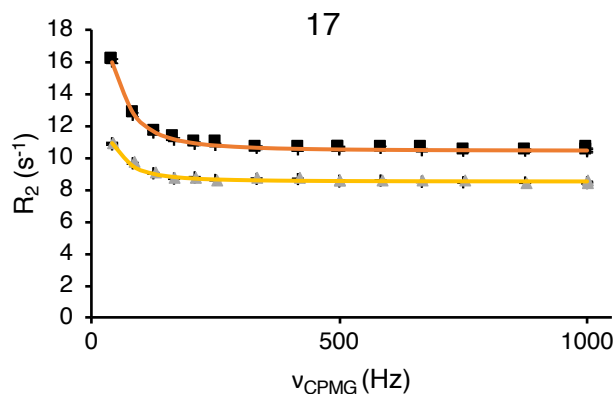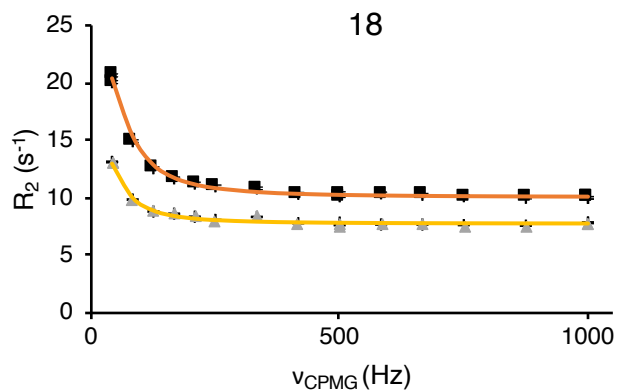

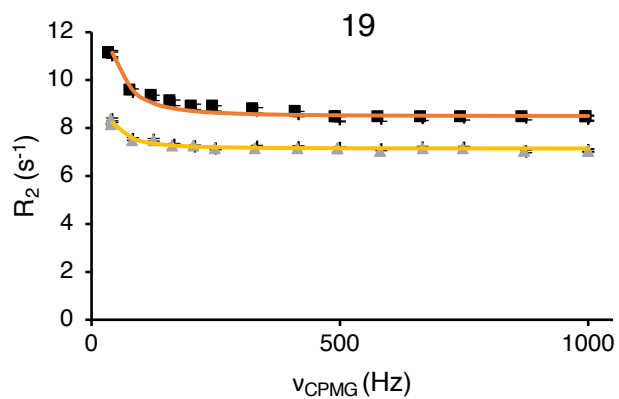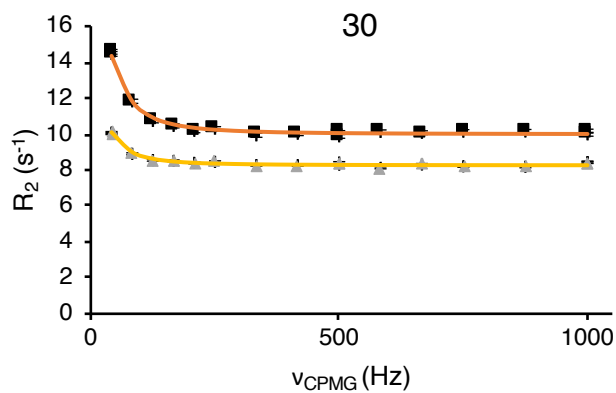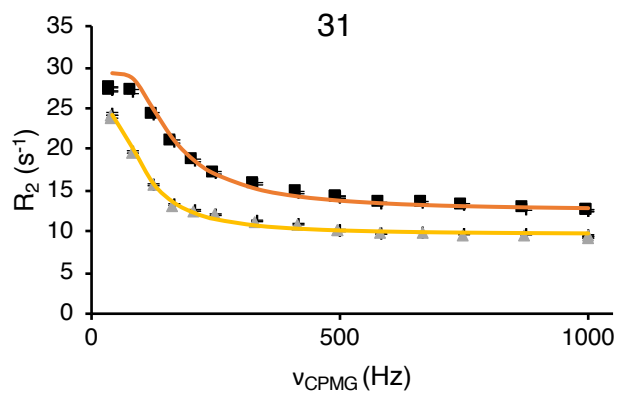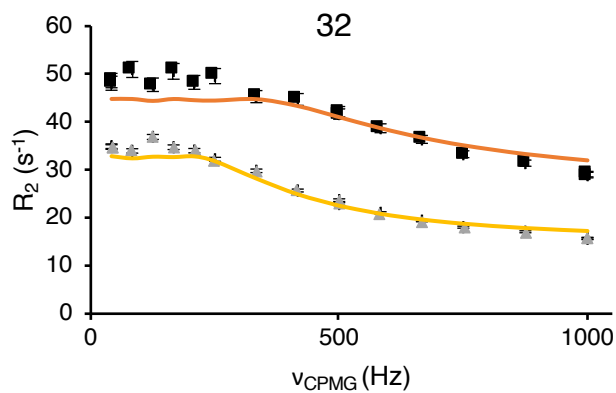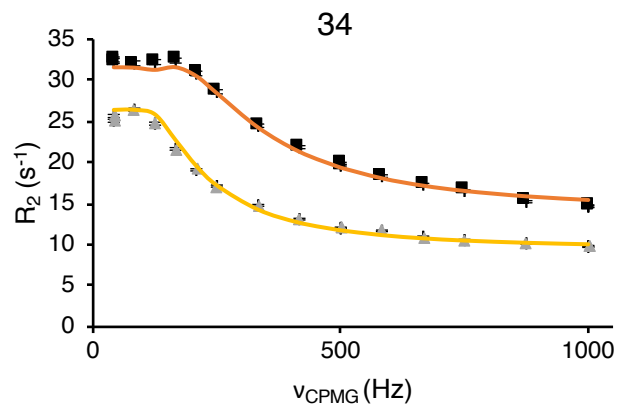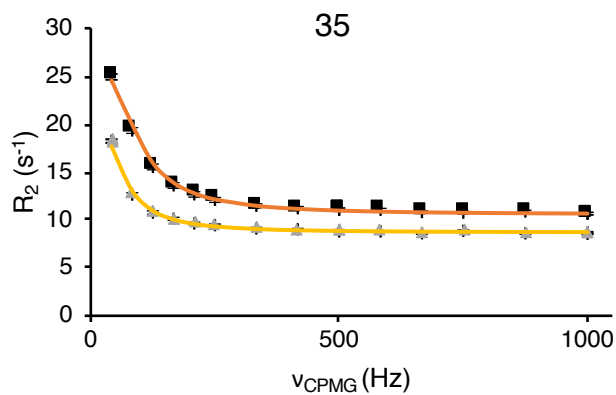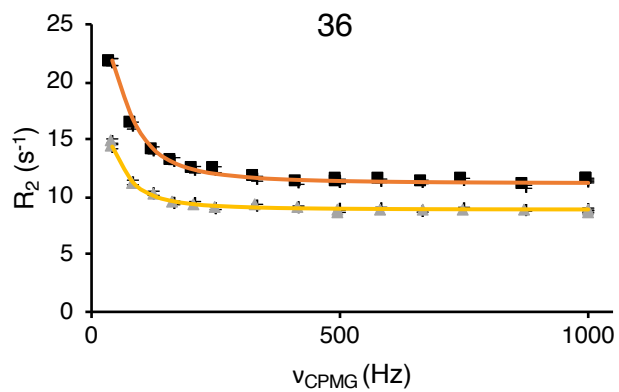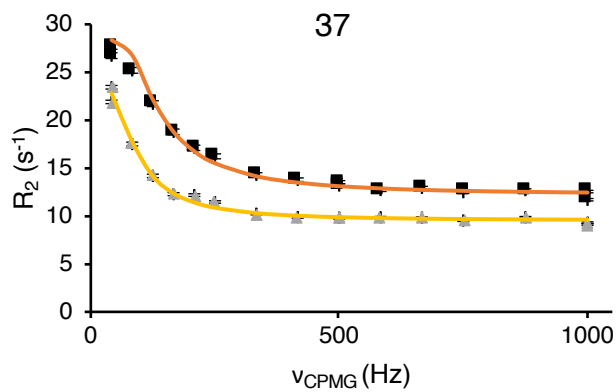

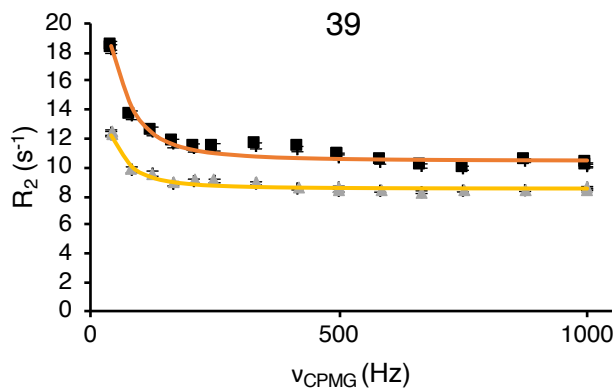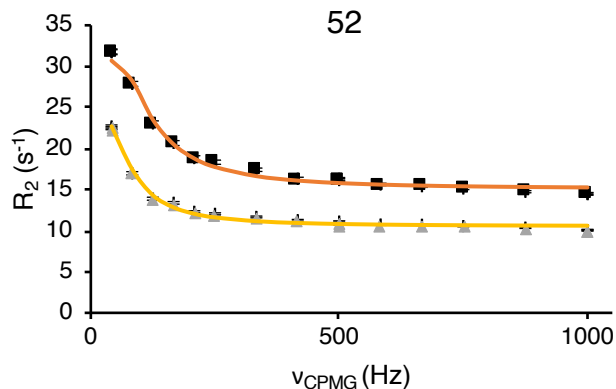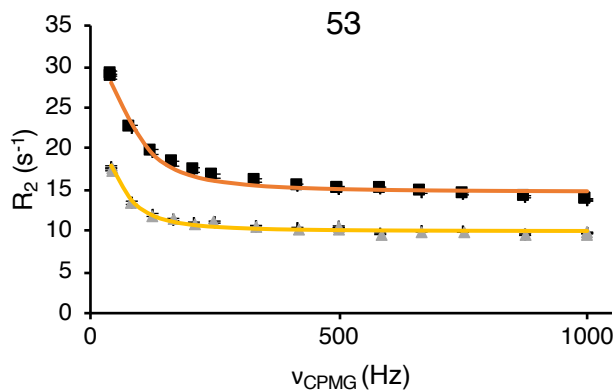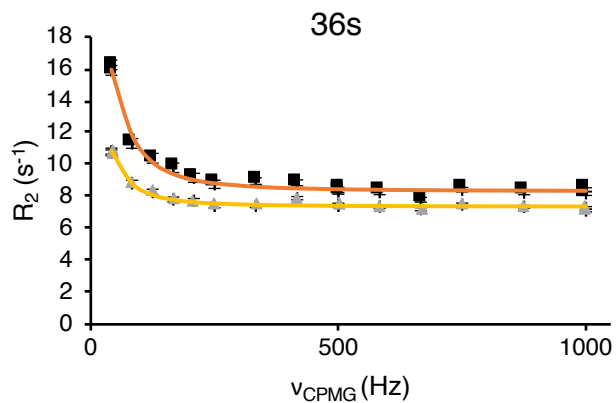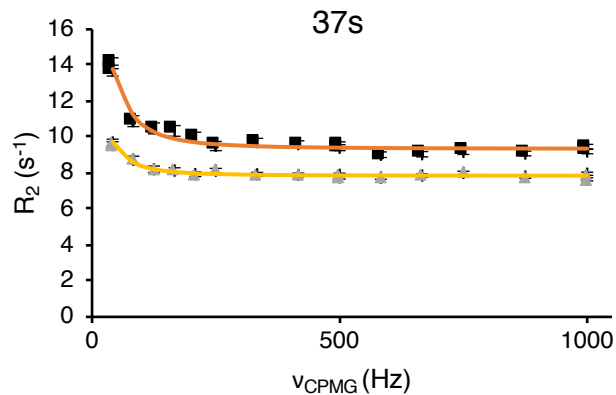

Supplement: S20 Fig — The top of each plot is labeled with the residue number, and 36s and 37s refer to the tryptophan sidechain NH groups. (PDF) [file pcbi.1007815.s024.pdf]
