# Supplementary figures and images for: Correction: A disordered encounter complex is central to the yeast Abp1p SH3 domain binding pathway
Source: PLoS Comput Biol. 2025 Jun 18;21(6):e1013201. doi: 10.1371/journal.pcbi.1013201 (PMC12176205; doi:10.1371/journal.pcbi.1013201)

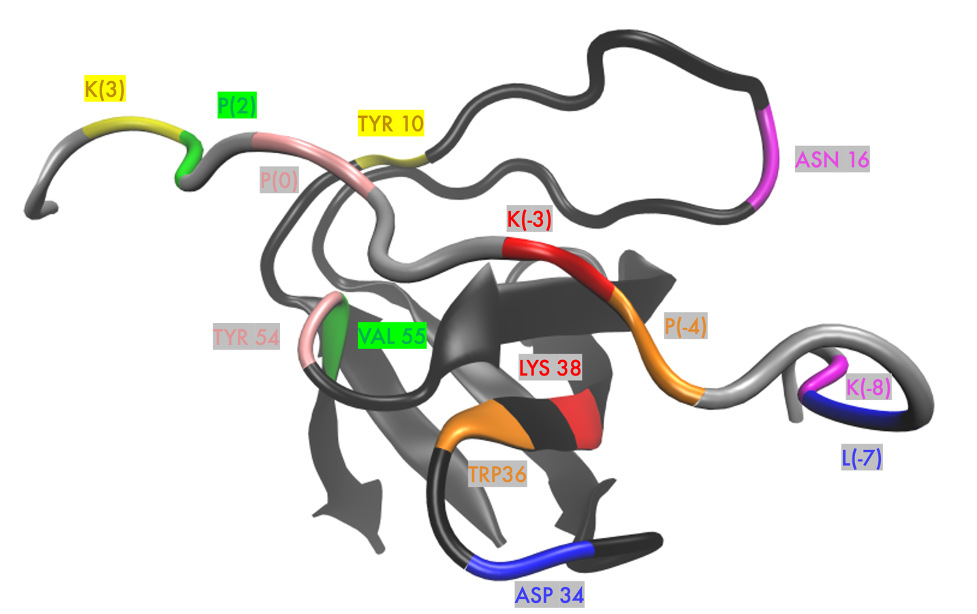

Supplement: S1 Fig — The distances K(3)-Y10, P(2)-V55, P(0)-Y54, and K(-3)-K38 were used in the seg1 simulations and all seven distances were used in the ArkA simulations. The pairwise distance in the NMR ranges from 8.73 to 8.99 Å. (PNG) [file pcbi.1013201.s001.png]
